# South West Orthopaedic Club, Spring 1990 Meeting

**Published:** 1990-12

**Authors:** 


### South West Orthopaedic Club

### Spring Meeting in Torbay 1990

### FAILURE OF A TOTAL KNEE REPLACEMENT DUE TO HYPERSENSITIVITY TO THE FEMORAL COMPONENT ALONE

**O. Thomas and S. Sampath**
Royal Gwent Hospital, Newport

A forty year old woman, with a history of metal allergy, had a technically correct Install Burstein Knee replacement for osteoarthritis. This prosthesis consists of a Cobalt-Chrome femoral component, and Titanium alloy! tibial tray. She rapidly developed pain and a decreasing range of motion. A technEtium bone scan was hotter over the femoral component. The knee was explored and a synovial biopsy showed changes compatible with a hypersensitivity reaction. Aerobic and anaerobic cultures were negative. An arthrodesis using Stainless Steel pins was done for intractable symptoms. She developed cellulitis around all four pin sites. S. Aureus was cultured from one site, but she failed to settle on appropriate antibiotics. The pins were removed and she settled fully after two weeks.

Battery patch testing showed hypersensitivity to nickel and cobalt, both found in the femoral component and pins only.

We believe that this patient lost her total knee replacement due to a hypersensitivity reaction to the femoral component alone.

We suggest that patients should be asked about metal allergies before choosing the implant and that it should be borne in mind that hypersensitivity may occur to one component only.

We further suggest that in a technically correct knee replacement, a progressive early decrease in the range of motion with pain, in the absence of infection, may be due to a hypersensitivity reaction.

### THE TREATMENT OF DISPLACED SUBCAPITAL FRACTURES WITH THE EXETER HEMIARTHROPLASTY

**M. F. Pearse, S. Bande, K. O'Dywer, R. S. M. Ling**
Royal Devon and Exeter Hospital

Between April 1983 and march 1989 111 Exeter bipolar hemiarthroplasties were performed. Patients were selected on the basis of their preoperative mobility with 67.5% walking independently and 23.5% using 1 stick. Although the patients were elderly, average age 75 years, and often had associated medical problems (68.5%) the operation was well tolerated. At least 70% were discharged back to their previous abode by 6 weeks and the mortality at 6 months was only 6.3%.

Seventy-four patients were reviewed with an average follow up of 38 months. 82.5% had a good or excellent result.

There were 3 cases of interprosthetic dislocation, all with an early version of the prostthesis and 2 cases of severe hip pain of unknown cause requiring conversion to a total hip replacement.

There was no clinical or radiological evidence of any significant acetabular erosion. The interprosthetic movement was assessed radiographically in 37 patients. 14 had more than 5° of movement within the prosthesis.

The results with this prosthesis are encouraging and concern that acetabular erosion will inevitably develop have not been confirmed. In a significant proportion the prosthesis continues to function as a true bipolar after an average period of 33 months.

We will continue to use the Exeter bipolar hemiarthroplasty to treat acute displaced subcapital femoral neck fractures in the mobile elderly patient.

### THE USE OF THE DICK PEDICULAR FIXATION DEVICE IN SPINAL TRAUMA

**T. Green**
Gloucester

Over a two year period all patients who sustained unstable spinal fractures following trauma and who were treated with the use of a Dick's Pedicular Screw Spinal Fixation Device, were reviewed. The indications for the use of such a device and a brief guide to the technique are discussed with a reveiw of the results in each case.

The Dick Spinal Fixator is shown to be a versatile and straight-forward method of producing fixation in the presence of unstable spinal fractures, allowing for early mobilisation, preservation of spinal anatomy, and healing of the original injury.

### THE USE OF INJURY SEVERITY SCORING FOR TRAUMA AUDIT

**A. J. Ward, E. J. Smith and I. J. Leslie**
Bristol Royal Infirmary

A clinical audit of acute trauma patients is presented to highlight the uses and limitations of injury severity scoring in the evaluation of trauma care. All 1676 paediatric and 893 adult trauma patients admitted with 24 hours of injury to Bristol Royal Infirmary were studied during the six month period, January to June 1989.

Revised Trauma Score (TRS) determinants were recorded in the Accident Department on proforma and the Injury Severity Score (ISS), derived from the Abbreviated Injury Scale (AIS-85), calculated on discharge or death in all cases. The Paediatric Trauma Score (PTS) was also recorded for paediatric patients.

A Trauma Register was compiled on computer database and TRISS methodology was used to calculate the probability of survival based on RTS, ISS, age and mechanism of injury (blunt or penetrating). Pre-Charts were constructed to illustrate the predicted and actual outcomes in severely injured or fatality cases. There were 27 deaths (2.5% of all acute admissions). The DEFinitive method was usd to calculate Z, W and M Statistics for comparison of outcome and severity of these cases with those of other institutions and "norm" data.

The value of injury scoring in trauma audit is shown and inadequacies of the current methods regarding the assessment of multiple skeletal injured and elderly patients are discussed.

### PATHOGENESIS AND TREATMENT OF GANGLIA

**A. Fowler**
Bridgend

A ganglion is a "blow out" from either a joint cavity or from the cavity of a synovial sheath. Fluid is pumped into the cavity of the ganglion and there is a one way return valve mechanism which prevents it going back.

For this reason all ganglia arising from joints extend down to the joint capsule and where there are overlying tendons, such as on the back of the wrist, the gaglion has a deep portion beneath the tendons and a superficial portion beneath the skin.

If excision involves the removal of only the superficial portion or if the valve mechanism in the joint capsule is not either excised or closed then the ganglion may recur. This is the cause of the high recurrence rate when the ganglion is treated as a superficial lesion.

This mechanism of ganglion formation also explains why, in some cases, the ganglion does *not* recur after incomplete removal. This is due to the fact that where a valve mechanism is due to the operation of a flap or the Bunsen principle, the valve will not begin to operate until a certain amount of pressure has been built up on the other side of the valve. Hence decompression by either rupture, needling or partial excision of the ganglion may result in cure.

### A FRACTURE OF THE PEDICLE OF L4 ASSOCIATED WITH A CONTRALATERAL SPONDYLOLISIS

**C. Weatherley, L. Vanden Berghe and H. Mehdian**
Princess Elizabeth Orthopaedic Hospital, Exeter

A unilateral pars defect of a lower lumbar vertebra leads to increased stress on the contralateral pedicle. This may result in a stress fracture of the pedicle. The condition is rare, but may be a cause of chronic back pain.

A twenty six year old physical education instructor with a 16 month history of back and left leg pain was found to have a unilateral spondylolisis and contralateral pedicle fracture of L4. This was treated by screw fixation and bone grafting of both defects with immediate relief of symptoms. At six months the patient remains symptom free and a CT scan confirms a soldi fusion.

The purpose of this report is to draw attention to a rarely reported condition and to commend a technique of instrumentation and fusion which involves only the symptomatic level.

### ARTHROSCOPIC MANAGEMENT OF TIBIAL PLATEAU FRACTURES

**Kevin J. O'Dwyer and Vladmir R. Bobic**
Princess Elizabeth Orthopaedic Hospital, Exeter

Seven cases of tibial plateau fractures were arthroscoped. In six patients, internal fixation was performed under arthroscopic control whilst in the one other case, washout alone was the only treatment deemed necessary. Post oeprative regime consisted of knee movements and quadriceps exercises commenced in the immediate postoperative period and in only one patient was any form of external splintage used. In all cases a good result was obtained. We propose that arthroscopic treatment is a useful adjunct to treatment of certain tibial plateau fractures as it combines the benefits of internal fixation without many of the risks.

### THE PATTERN OF NATIONAL HUNT INJURIES

**G. D. Rooker**
Cheltenham

National Hunt racing is divided into 2 sections. Hurdle races are run over small objects at a fast pace by young horses as opposed to steeple chases which are run by older horses at a slower pace over large obstacles. On average each National Hunt jockey may expect to fall once in every 11 rides and be injured sufficiently to prevent him riding once in every 7 falls. Injury patterns have been reviewed between 1984 and 1988, during which time there has been no significant increase in the number of injuries and no major change in their pattern. The figures have been obtained from the Injured Jockeys Compensation Fund and do not cover amateur riders. The majority of injuries are incurred around the shoulder region (70%) with the wrist and tibia accounting for a significant proportion of the remainder. The injury patterns have been correlated with the fashion in which a jockey falls, always trying to land on his shoulder blade with this fact accounting for the relatively few injuries in this extremely hazardous sport.

The Gary Hampson Memorial Prize was awarded to Mr V. G. Langkamer who gave a lecture entitled "The effects of Low Modulus Femoral Prosthesis on the Strain Distribution and Magnitude in the Proximal Femur and the Relationship to Bone Remodelling." The X-ray Quiz was won by Mr A. J. Ward of Bristol. The S. W. Orthopaedic Club Prize Certificate was awarded to Mr T. Green of Gloucester and the presentation was made by Mr Karl Nissen.

