# Arthroscopic Management for Tibial Plateau Fractures

**Published:** 1991-12

**Authors:** 


**ADDENDUM —**
**Correction to Report of S.W. Orthopaedic Club Torbay Meeting in W.E.M.J. Vol. 105(iv) p.128.**
The S.W. Orthopaedic Club Prize for the best paper was awarded to Mr. K. J. O'Dwyer for his paper entitled "Arthroscopic Management for Tibial Plateau Fractures".

